# STiLE: automated tissue microarray dearraying for spatial transcriptomics

**DOI:** 10.1093/bioinformatics/btag572

**Published:** 2026-10-06

**Authors:** Harsh Sinha, Arun Das, Yu-Chiao Chiu, Shou-Jiang Gao, Yufei Huang

**Affiliations:** Intelligent Systems Program, University of Pittsburgh, Pittsburgh, PA, United States; Cancer Virology Program, UPMC Hillman Cancer Center, Pittsburgh, PA, United States; Department of Medicine, University of Pittsburgh School of Medicine, Pittsburgh, PA, United States; Department of Medicine, University of Pittsburgh School of Medicine, Pittsburgh, PA, United States; Department of Computational and Systems Biology, University of Pittsburgh School of Medicine, Pittsburgh, PA, United States; Cancer Therapeutics Program, UPMC Hillman Cancer Center, Pittsburgh, PA, United States; Cancer Virology Program, UPMC Hillman Cancer Center, Pittsburgh, PA, United States; Department of Microbiology and Molecular Genetics, University of Pittsburgh School of Medicine, Pittsburgh, PA, United States; Intelligent Systems Program, University of Pittsburgh, Pittsburgh, PA, United States; Cancer Virology Program, UPMC Hillman Cancer Center, Pittsburgh, PA, United States; Department of Medicine, University of Pittsburgh School of Medicine, Pittsburgh, PA, United States; Department of Electrical and Computer Engineering, University of Pittsburgh, Pittsburgh, PA, United States

## Abstract

**Summary:**

Tissue microarrays (TMAs) enable high-throughput spatial transcriptomic profiling of dozens of tissue cores on a single slide. However, existing dearraying methods operate on histological images and do not support the coordinate-based outputs of spatial transcriptomics platforms. Therefore, task of assigning cells to their respective cores (dearraying) remains a manual bottleneck. We present STiLE, a tool for automated TMA dearraying that operates solely on cell centroid coordinates. By eliminating dependence on image data, STiLE is robust to artefacts such as variable staining quality and uneven illumination. The algorithm combines connectivity-based component detection, density-based clustering (HDBSCAN), component-guided cluster merging, and optional grid-based peak detection. Validation on seven public TMA samples (50–150 cores, three platforms) achieved ARI > 0.99, while systematic benchmarking on 396 synthetic datasets with realistic artefacts demonstrated consistently robust performance (mean ARI = 0.992). STiLE accepts standard formats (AnnData, CSV) and is platform-agnostic, supporting diverse platforms including Vizgen MERSCOPE, 10× Xenium, and NanoString CosMx. An interactive Streamlit interface enables parameter tuning, visual inspection, and region-based processing for large slides.

**Availability and implementation:**

https://pypi.org/project/stile; source at https://github.com/Huang-AI4Medicine-Lab/stile

## 1 Introduction

Imaging-based spatial transcriptomics (iST) platforms such as 10× Xenium (Janesick *et al.* 2023), NanoString CosMx (He *et al.* 2022), and Vizgen MERSCOPE (Chen *et al.* 2015) enable simultaneous profiling of hundreds to thousands of RNA targets *in situ* at subcellular resolution while preserving tissue architecture. These technologies provide unprecedented insight into spatial gene expression programs, tumour microenvironments, and cellular interactions. However, their high per-slide cost and limited field of view make large-cohort studies expensive and logistically challenging.

To address this constraint, many studies adopt tissue microarray (TMA) experimental designs (Kononen *et al.* 1998, Meng *et al.* 2025), in which dozens to hundreds of tissue cores from different patients are arrayed onto a single slide. When combined with iST, TMAs enable cohort-scale spatial profiling while amortizing imaging cost across many specimens (Wang *et al.* 2025). This strategy generates datasets containing millions of spatially resolved cells across large patient populations.

A critical preprocessing step in TMA-based iST is dearraying: assigning each segmented cell to its source core before downstream analysis. Accurate core assignment is essential for per-patient analyses, differential expression, spatial statistics, and integrative clinical modelling. Despite its importance, dearraying remains a practical bottleneck.

Some vendors provide platform-specific software with ROI-oriented annotation functionality but these tools typically require users to define cores manually. This manual approach can be practical for a smaller TMA slide, but it becomes cumbersome for large TMA slides containing hundreds of cores. An automated dearraying approach is therefore required to segment and assign cores reproducibly.

Existing dearraying approaches have been developed primarily for histological and multiplexed protein imaging workflows. Tools such as QuPath (Bankhead *et al.* 2017), ATMAD (Nguyen *et al.* 2018), Coreograph within MCMICRO (Schapiro *et al.* 2022), TMA-Grid (Ge *et al.* 2024), and PRISM (Tubelleza *et al.* 2025) operate on raster images, detecting core boundaries through morphological features or deep learning-based segmentation. These methods assume dense, spatially continuous images with consistent tissue-background contrast, as produced by brightfield histology or multiplexed immunofluorescence platforms (e.g. CODEX, CyCIF, mIHC).

However, iST platforms differ fundamentally in both data structure and analytical workflow. Although fluorescence images are acquired during assay processing, standard downstream analysis relies on coordinate-based outputs consisting of cell centroids with associated transcript counts. Moreover, the fluorescence signals in iST are sparse and marker-dependent, lacking the uniform tissue-background contrast expected by existing image-based dearraying algorithms. In many experimental settings, matched histology images from the same slide may not be available, making image-driven TMA dearraying infeasible. Applying existing tools would require reverting to raw image data, reintroducing sensitivity to staining variability, uneven illumination, & tissue artefacts, despite the fact that coordinate data alone contains sufficient geometric information to delineate cores.

Furthermore, most image-based methods assume a regular grid layout, an assumption that breaks down when cores are misaligned, variably spaced, partially damaged, or missing. Grid drift and non-uniform spacing are common in large TMAs, particularly in slides subjected to sectioning artefacts or deformation. These limitations highlight a methodological gap: no existing dearraying framework is designed to operate directly on coordinate-based spatial transcriptomics outputs.

To address this need, we developed STiLE (Spatial Tissue microarray Labelling and Extraction), a Python tool that performs automated TMA dearraying directly from cell centroid coordinates. By operating entirely in coordinate space, STiLE eliminates dependence on histological images and is intrinsically robust to staining variability, illumination artefacts, and platform-specific imaging characteristics. Rather than imposing a rigid grid model, STiLE leverages connectivity-based component detection and density-based clustering to identify spatially coherent cores based on intrinsic geometric structure. A grid-aware refinement step regularizes core positions when grid structure is present, while preserving flexibility under irregular layouts.

STiLE is an automated, platform-agnostic tool that integrates directly with AnnData-based workflows, and scales to million-cell datasets. This enables reproducible dearraying without requiring users to manually select each core within vendor-specific software. To our knowledge, it is the first dearraying method designed specifically for coordinate-based spatial transcriptomics data. By removing a persistent preprocessing bottleneck, STiLE enables scalable, cohort-level spatial transcriptomic analysis using cost-efficient TMA experimental designs.

## 2 Implementation

STiLE operates through a modular, multi-phase pipeline designed to robustly separate spatially coherent TMA cores using only cell centroid coordinates (see Section S1, available as supplementary data at *Bioinformatics* online). It consists of four stages: connectivity analysis, density-based clustering, and optional grid-based refinement (Fig. 1).

**Figure 1:**
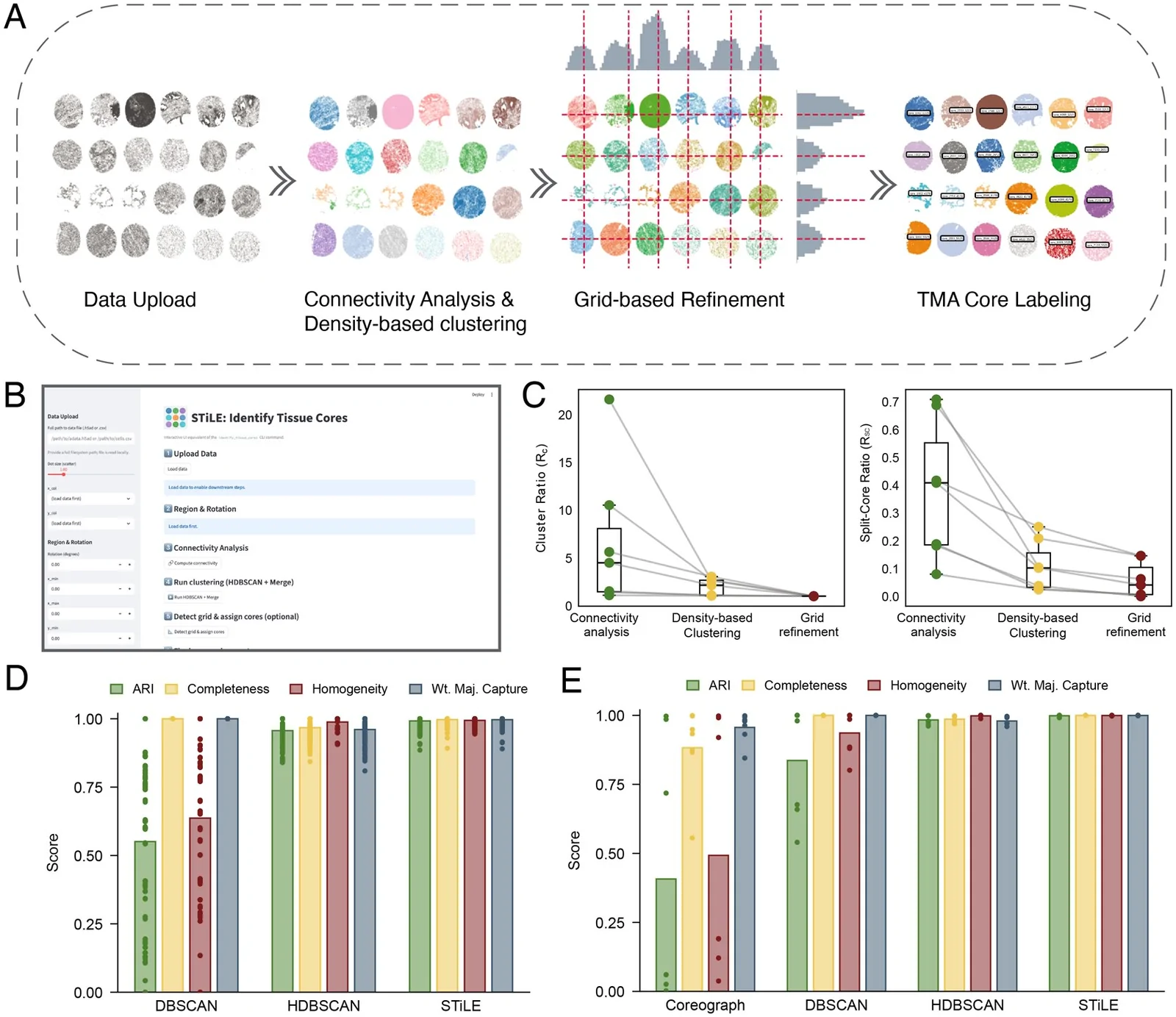
Overview of the STiLE dearraying workflow, interactive interface, and robustness evaluation. (A) Algorithmic pipeline. Cell centroids from spatial transcriptomics TMA data (CSV or AnnData) are first grouped into spatially coherent clusters via connectivity analysis on buffered geometric unions, followed by density-based clustering (HDBSCAN). An optional grid-based refinement step detects peaks in marginal density histograms to regularize core positions, after which cells are reassigned to their nearest core center. For large slides, the pipeline can be applied to sub-regions independently and merged. (B) Streamlit-based interactive interface for parameter adjustment, real-time visualisation, validation, and sub-region processing. (C) Ablation analysis of STiLE components. Performance was evaluated across seven real TMA datasets by sequentially adding connectivity analysis, density-based clustering, and grid refinement. Over-segmentation was quantified using the Cluster Ratio ($R_{C}$; ideal value = 1) and Split-Core Ratio ($R_{SC}$; ideal value = 0). (D–E) Systematic robustness assessment of Coreograph, DBSCAN, HDBSCAN, and STiLE evaluated on simulated (D) and real (E) datasets, quantified by four metrics: Adjusted Rand Index (ARI), completeness, homogeneity, and weighted majority capture (higher values indicate better performance). Bars show the mean across datasets; overlaid points show individual datasets. Coreograph shows the lowest and most variable accuracy, DBSCAN shows variable ARI across datasets, and HDBSCAN and STiLE achieve consistently high scores on all four metrics.

### 2.1 Connectivity analysis

The first phase identifies spatially separated coarse regions that correspond to candidate TMA cores. Each cell centroid is expanded by a buffer radius *r*, and cells whose buffered regions overlap are connected to form an undirected graph. Two circular buffers centred at points $p_{i}$ and $p_{j}$ overlap if and only if $|p_{i}-p_{j}|\leq 2r$. Edges are computed efficiently using KD-tree-based neighbour queries, returning all pairs (i,j) satisfying the distance constraint in O(n  log  n+|P|) time, where |P| is the number of proximal pairs. The resulting sparse adjacency matrix is traversed using breadth-first search to extract connected components.

The default buffer radius *r* is defined as the median nearest-neighbour distance estimated from a random subsample of up to 50 000 cells. This statistic provides a robust estimate of the intrinsic intra-core spatial scale. Within a TMA core, cells are densely packed, so the median nearest-neighbour distance captures typical intra-core spacing. In contrast, inter-core gaps are typically substantially larger than 2*r*, ensuring that cells from distinct cores fall into separate connected components. Users may override the default radius in rare cases where inter-core gaps approach intra-core spacing (e.g. nearly touching cores).

### 2.2 Density-based clustering

Connectivity analysis separates spatially disconnected regions but may retain sparse debris or segmentation artefacts within components. To refine core structure, STiLE applies HDBSCAN (Hierarchical Density-Based Spatial Clustering of Applications with Noise) with leaf cluster selection independently within each connected component (McInnes *et al.* 2017). Leaf-cluster selection favors compact, high-density clusters and reduces the risk of merging adjacent cores connected through sparse bridges. Noise points, including debris and low-density inter-core cells, are labelled as outliers and excluded from initial core assignments. Because tissue folding, partial sectioning, or density gradients may cause HDBSCAN to partition a single biological core into multiple subclusters. STiLE resolves this by merging all clusters that share a connected component from the first phase. This component-guided merging restores biologically coherent cores while preserving the noise filtering benefits of density-based clustering. For well-separated TMAs, this step typically yields final core assignments without further processing.

### 2.3 Grid-based refinement

In challenging cases, such as sparse cores, fragmented tissue, or narrow inter-core gaps, connectivity analysis alone may yield fragmented or partially merged cores. To address this, STiLE provides an optional grid-aware refinement step that leverages global array structure without imposing a rigid grid assumption. Specifically, cell coordinates are projected onto marginal density histograms along the *x* and *y* axes (Fig. 1A). Peaks are detected using prominence-based criteria, ranked by the product of peak prominence and histogram height. The top $k_{x}$ and $k_{y}$ peaks define candidate grid coordinates along each axis. The cartesian product of these peak sets yields candidate core centers. Merged clusters are then reassigned to their nearest candidate center. Candidate centers with insufficient support (below a minimum cell count threshold) are discarded. This hybrid approach preserves the adaptability of connectivity clustering while leveraging the grid structure when available.

### 2.4 Interactive interface

For large slides with heterogeneous spacing, users may define rectangular sub-regions, process each independently, and merge results with region-specific labels (see Section S2, available as supplementary data at *Bioinformatics* online). Apart from python-based programmatic interface, STiLE also provides a Streamlit-based web application that can be deployed locally or on institutional servers, avoiding external data transfer. It supports the complete workflow, including parameter selection, data upload, region selection, parameter adjustment for each phase, real-time visualisation, and export of annotated results (see Section S3, available as supplementary data at *Bioinformatics* online).

## 3 Results

We validated STiLE on publicly available TMA datasets from Wang *et al.* (2025), comprising tumour and normal tissue arrays profiled on three imaging-based spatial transcriptomics platforms: 10× Xenium, NanoString CosMx, and Vizgen MERSCOPE. Ground truth core identities were derived from the original TMA layout maps. Because STiLE assigns arbitrary core indices that do not correspond to ground truth labels, we used the Hungarian algorithm to find the optimal one-to-one matching between predicted and true core assignments before computing evaluation metrics. Dataset-specific parameter tuning was required, reflecting differences in core spacing, core fragmentation, and array geometry across platforms and samples (see Table S1, available as supplementary data at *Bioinformatics* online).

We compare STiLE against coordinate-based clustering baselines, including HDBSCAN (McInnes *et al.* 2017), DBSCAN (Ester *et al.* 1996), as well as Coregraph, an image-based dearraying method (Schapiro *et al.* 2022). Coreograph requires fluorescence images as input, so the comparative evaluation was restricted to the seven datasets for which corresponding fluorescence images were available. All methods were evaluated using four accuracy metrics: ARI, completeness, homogeneity, and weighted majority capture (see Fig. 1E). We also assessed over-segmentation using two metrics: Cluster Ratio ($R_{C}$) and the Split-Core Ratio ($R_{SC}$) (see Fig. S1, available as supplementary data at *Bioinformatics* online).

Coreograph showed the lowest overall accuracy with a mean ARI of 0.4, and frequently failed to recover all cores ($R_{C}\ll 1$). HDBSCAN achieves high accuracy but consistently over-segmented cores with RC values up to 2.0. DBSCAN tends to merge some cores ($R_{C}<1$), thus exhibiting substantially greater variablility in accuracy across datasets, with ARI values ranging from 0.5 to 0.9. In contrast, STiLE achieves consistent scores with near-perfect accuracy and minimal over-segmentation ($R_{C}=1$, and low $R_{SC}$) across all datasets.

We perform an ablation study to quantify the contribution of STiLE’s three major components, namely connectivity-analysis, density-based clustering, and grid refinement (Fig. 1C). Connectivity analysis alone provides an initial partition of the TMA cores but results in substantial over-segmentation, with a median $R_{C}$ of 4.5 and a median $R_{SC}$ of 0.41. Adding density-based clustering accounts for the largest reduction, lowering the median $R_{C}$ to 2 and the median $R_{SC}$ to 0.10. Grid refinement resolves the residual cases, bringing the median $R_{C}$ to 1 and the median $R_{SC}$ below 0.05. Both metrics decrease at each successive stage across all the seven datasets, demonstrating that each component makes a distinct and measurable contribution to reducing over-segmentation.

To systematically assess robustness under distorted, incomplete and heterogenous cores, we generated 396 synthetic TMA datasets with realistic artefacts (see Section S4, available as supplementary data at *Bioinformatics* online). The simulations varied with three primary parameters: core size irregularity, cell density bias across cores and core missingness. All synthetic datasets additionally included elliptical core deformation, tissue artefacts (slits, bubbles, folds), affine warping, and non-rigid thin-plate-spline deformation to mimic real-world variability. Across all 396 configurations, STiLE achieved a mean ARI of 0.992 (median 1.000, minimum 0.885), with 99.7% of datasets exceeding ARI 0.90 (Tables S6–S8, available as supplementary data at *Bioinformatics* online). Performance remained stable under challenging conditions: mean ARI was 0.991 even at 100% radius jitter, and degradation was minimal with up to 50% missing cores. These results demonstrate that STiLE’s connectivity-based approach is robust to the geometric and density variations commonly encountered in real TMA preparations.

Processing time scaled approximately as O(n  log  n) with cell count *n*, dominated by HDBSCAN clustering and kd-tree construction; datasets with up to one million cells were completed within a couple of minutes on standard hardware (see Table S2, available as supplementary data at *Bioinformatics* online).

## 4 Discussion

STiLE resolves a persistent preprocessing bottleneck in TMA-based spatial transcriptomics by enabling automated dearraying directly from cell centroid coordinates. By operating entirely in coordinate space, it avoids dependence on histological image quality, staining variability, and platform-specific imaging, unlike image-based methods such as MCMICRO (Schapiro *et al.* 2022) and ATMAD (Nguyen *et al.* 2018). This makes STiLE immediately applicable to platforms including 10× Xenium, NanoString CosMx, and Vizgen MERSCOPE without retraining or image preprocessing.

Its modular design adapts to diverse TMA configurations. For well-separated cores, connectivity analysis with density-based clustering yields accurate assignments. For dense or irregular arrays, optional grid-based refinement introduces global structural guidance without enforcing a rigid grid. Region-based processing further supports large slides with heterogeneous spacing, allowing flexibility while maintaining robustness. Unlike end-to-end systems such as PRISM (Tubelleza *et al.* 2025), STiLE focuses on dearraying as a modular component, producing interoperable outputs that integrate into existing workflows while eliminating a manual preprocessing step.

STiLE assumes that upstream cell segmentation is reliable and it uses the resulting spatial coordinates to identify TMA cores. The strategy further assumes that inter-core gaps exhibit lower cell density than intra-core regions. As a result challenging cases such as touching cores, extremely sparse cores, or cores split into disconnected fragments may require parameter tuning and, in some cases, manual review or region-based processing using STiLE’s interactive interface.

Overall, by reframing TMA dearraying as a geometric clustering problem in coordinate space, STiLE provides automated, scalable and platform-agnostic solution for cohort-scale spatial transcriptomic analysis.

## Author contributions

Harsh Sinha (Conceptualization [Lead], Data curation [Lead], Investigation [Lead], Methodology [Lead], Software [Lead], Validation [Lead], Visualization [Lead], Writing—original draft [Lead], Writing—review & editing [Equal]), Arun Das (Conceptualization [Lead], Data curation [Supporting], Writing—original draft [Equal], Writing—review & editing [Equal]), Yu-Chiao Chiu (Funding acquisition [Equal], Writing—review & editing [Equal]), Shou-Jiang Gao (Funding acquisition [Equal], Writing—review & editing [Equal]), and Yufei Huang (Conceptualization [Lead], Funding acquisition [Equal], Supervision [Lead], Writing—original draft [Equal], Writing—review & editing [Equal])

## Supplementary Material

btag572_Supplementary_Data

## Data Availability

No new experimental data were generated for this study. Public TMA datasets used for validation were obtained from (Wang *et al.* 2025) [10.5281/zenodo.16847475]. Simulated TMA datasets and STile-generated core annotations for all public datasets are available at Zenodo [10.5281/zenodo.18880122]
