# Supplementary material for: STiLE: automated tissue microarray dearraying for spatial transcriptomics
Source: Bioinformatics. 2026 Oct 6;42(10):btag572. doi: 10.1093/bioinformatics/btag572 (PMC13652026; doi:10.1093/bioinformatics/btag572)
Supplement: Supplementary file 1 [file btag572_supplementary_data.pdf]

Supplementary Materials

STiLE: Automated Tissue Microarray Dearraying
for Spatial Transcriptomics

Harsh Sinha, Arun Das, Yu-Chiao Chiu, Shou-Jiang Gao, Yufei Huang

Contents

1 Algorithm Details **2**
1.1 Pipeline Pseudocode 2
2 Region-Based Workflow **2**
3 Interactive Interface **3**
3.1 Programmatic Usage 3
4 Results **4**
4.1 Processing Time 4
4.2 Evaluation on TMA datasets 4
4.3 Simulation Benchmark 4
5 Data Format Conventions **6**
5.1 Input Requirements 6
6 Parameter Reference **6**
6.1 Output Format 6
7 Evaluation Metrics **7**

S1 Algorithm Details

Algorithm S1: STiLE Pipeline

Input: AnnData object `adata` with cell coordinates (x_i, y_i) for $i = 1, \ldots, N$
Output: Core assignment labels ℓ_i for each cell

// Phase 1: Connectivity analysis
$d_{\text{med}} \leftarrow$ MedianNNDist($\{(x_i, y_i)\}$, k=2, subsample=50000)
$r \leftarrow d_{\text{med}}$ // buffer radius (user-overridable)
$\mathcal{T} \leftarrow$ cKDTree($\{(x_i, y_i)\}$)
$\mathcal{P} \leftarrow \mathcal{T}$.query_pairs($2r$) // all pairs within $2r$
$\mathbf{A} \leftarrow$ SparseAdjacency($\mathcal{P}$)
$\{C_1, C_2, \ldots, C_K\} \leftarrow$ ConnectedComponents($\mathbf{A}$)

// Phase 2: HDBSCAN clustering
$\{h_i\} \leftarrow$ HDBSCAN($\{(x_i, y_i)\}$, `min_cluster_size`, `min_samples`, method='leaf')
// $h_i = -1$ denotes noise

// Phase 3: Component-guided merging
for *each component* C_k **do**
  if $|C_k| = 1$ ***or*** $\forall\, i \in C_k : h_i = -1$ **then**
    $m_i \leftarrow -1$ for all $i \in C_k$ // unassigned
  else
    $m_i \leftarrow k$ for all $i \in C_k$ // merge under component ID
  end
end
// Optional early exit if merged clusters $\approx$ cores

// Phase 4: Grid-based peak detection (optional)
$\mathbf{H}_x \leftarrow$ Histogram($\{x_i\}$, bins=B_x)
$\mathbf{H}_y \leftarrow$ Histogram($\{y_i\}$, bins=B_y)
$\mathcal{X} \leftarrow$ FindPeaks($\mathbf{H}_x$, prominence, distance)
$\mathcal{Y} \leftarrow$ FindPeaks($\mathbf{H}_y$, prominence, distance)
$\mathcal{G} \leftarrow \mathcal{X} \times \mathcal{Y}$ // candidate grid centers
for *each merged cluster* $m_k \neq -1$ **do**
  $\bar{c}_k \leftarrow$ Centroid($\{(x_i, y_i) : m_i = k\}$)
  $g^* \leftarrow \arg\min_{g \in \mathcal{G}} \|\bar{c}_k - g\|$
  $\ell_i \leftarrow g^*$ for all i with $m_i = k$
end
Remove grid centers with $<$ `min_cells_per_center` assigned cells
Reassign affected clusters to remaining nearest centers

// Phase 5: Nearest-center reassignment (optional)
$\mathcal{T}_g \leftarrow$ cKDTree($\{$CoreCentroids$\}$)
for *each unassigned cell* i *(ℓ_i = unassigned)* **do**
  $\ell_i \leftarrow \mathcal{T}_g$.query($(x_i, y_i)$) // nearest core
end
return $\{\ell_i\}$

S1.1 Pipeline Pseudocode

Algorithm S1 provides a formal description of the STiLE pipeline. The five phases are applied sequentially, with Phases 4-5 being optional depending on data characteristics.

S2 Region-Based Workflow

For large TMA slides with many cores (>50), global peak detection on marginal density histograms can fail because:

1. **Non-uniform spacing**: Core pitch may vary across the slide due to manufacturing tolerances, causing peaks at inconsistent intervals.

Table S1: The parameter values used by STiLE to perform TMA dearraying for each dataset (12).

dataset_name	region_name	rotation_deg	region_x_min	region_x_max	region_y_min	region_y_max	buffer_distance	min_cluster_size	min_samples	num_rows	num_cols	density_bins_x	density_bins_y	peak_prominence	peak_distance	min_cells_per_center
2023_xenium_breast_nTMA	R1	0	0	20000	0	7100	21	100	7	4	6	200	200	15	15	3
	R2	5	0	20000	7100	12200	27	100	7	4	6	200	100	20	20	3
	R3	0	0	20000	12200	18000	60	100	7	4	6	200	100	20	20	3
2023_xenium_breast_tTMA1	R1	0	0	15000	0	6500	40	200	7	6	10	400	400	25	30	10
	R2	0	0	15000	6500	12500	40	200	7	6	10	400	400	25	30	10
	R3	0	0	15000	12500	18000	60	200	7	7	10	400	500	25	30	10
2023_xenium_lung_nTMA	R1	0	0	8000	0	7000	23	200	7	7	7	200	200	25	25	10
	R2	0	0	4100	7000	12100	9	200	7	3	3	100	200	25	30	10
	R3	0	4100	8000	7000	12100	45	200	7	3	3	200	200	25	30	10
	R4	0	0	8000	12100	16100	45	200	7	2	5	200	200	25	30	10
2023_xenium_lung_tTMA1	R1	0	0	15000	0	6500	60	200	7	6	10	400	500	25	30	10
	R2	0	0	15000	6500	12000	29	100	7	7	10	200	200	15	15	3
	R3	0	0	15000	12000	18000	19	100	7	7	10	200	200	15	15	3
2023_xenium_panhuman_nTMA	R1	0	0	8000	0	7000	45	200	7	4	6	200	200	25	25	10
	R2	0	0	8000	7000	12300	17	20	7	3	6	200	200	25	25	10
	R3	0	0	8000	12300	18000	60	20	7	2	6	200	200	25	25	10
2023_xenium_panhuman_tTMA1	R1	0	0	20000	13500	25000	15	100	7	7	10	200	200	15	15	3
	R2	0	0	20000	7500	13500	50	100	7	7	10	200	200	15	15	3
	R3	0	0	20000	0	7100	60	100	7	7	10	200	200	15	15	3
2024_xenium_breast_tTMA2	R1	0	0	15000	0	9000	27	200	7	5	6	400	500	25	30	10
	R2	0	0	15000	9000	18000	20	200	7	5	6	400	500	25	30	10

2. **Peak merging**: Adjacent cores may produce overlapping peaks in the marginal histogram, leading to under-detection.
3. **Missing cores**: Absent cores create gaps in the expected grid, breaking the assumption of uniform peak spacing.

STiLE's region-based workflow addresses these challenges:

1. **Region definition**: The user defines a rectangular bounding box $(x_{\min}, x_{\max}, y_{\min}, y_{\max})$ covering a subset of the TMA.
2. **Cropping**: Cells outside the bounding box are excluded. The cropped dataset retains original cell indices for later merging.
3. **Independent processing**: The full pipeline (Phases 1-5) is applied to the cropped region. Within each region, core spacing is locally consistent, enabling reliable peak detection.
4. **Region labelling**: Core labels are prefixed with the region name (e.g., `region1_core_1234_5678`) to prevent collisions across regions.
5. **Persistence**: Region results are saved as CSV files in a dataset-specific directory, enabling incremental processing across sessions.
6. **Merging**: After all regions are processed, a merge step combines results by matching cell indices and writing each region's prefixed core labels back to the original dataset.

The Streamlit interface provides visual guidance for region selection, displaying the current cell scatter plot with adjustable bounding box sliders.

S3 Interactive Interface

The Streamlit-based interface (`stile run`) provides a nine-step guided workflow:

1. **Data upload**: Load `.h5ad` or `.csv` files. For CSV files, users select the x and y coordinate columns. Optional rotation applies an affine transformation before processing.
2. **Region cropping**: Define rectangular bounds via sliders to restrict analysis to a sub-region. A scatter plot previews the selected region.
3. **Connectivity analysis**: Compute median nearest-neighbor distance and identify connected components. Displays the number of components found.
4. **HDBSCAN + merge**: Run HDBSCAN clustering and component-guided merging. Sidebar controls adjust `min_cluster_size` and `min_samples`. A scatter plot colored by merged cluster label provides immediate visual feedback.
5. **Grid detection**: Construct marginal density histograms and detect peaks. Interactive histograms with peak markers allow users to assess detection quality. Sidebar controls adjust prominence, distance, and bin count.
6. **Final assignment**: Review core assignments on a labeled scatter plot. Save the current region's results (with optional region name prefix).
7. **Region merging**: Select and merge previously saved regions into the full dataset.
8. **Export**: Download core assignments as CSV or save the annotated `.h5ad` file.
9. **Layout mapping**: Upload a layout CSV defining the desired core naming scheme (e.g., A01, A02, . . .). STiLE maps detected core positions to the layout grid and creates a `core_name` column.

All parameters persist across workflow steps via Streamlit session state. The interface supports iterative refinement: users can return to any step, adjust parameters, and re-run downstream steps.

S3.1 Programmatic Usage

For scripting and integration into custom workflows, the pipeline can be invoked directly from Python:

```
from stile.pipeline import identify_tissue_cores
import scanpy as sc

adata = sc.read_h5ad("data.h5ad")
adata = identify_tissue_cores(adata)
```

Individual phase functions (`find_connected_components`, `assign_cells_hdbscan`, `merge_clusters_by_component`, etc.) are importable from `stile.nodes` for fine-grained control.

S4 Results

S4.1 Processing Time

Table S2 reports wall-clock processing times on a standard workstation (Intel Xeon, 16 GB RAM, single-threaded except for HDBSCAN core distance computation with 4 threads).

Table S2: Processing time by dataset.

TMA	Platform	Cells	Time (s)	Phase Breakdown
Tumor	Xenium	1.2M	92	P1: 18s, P2: 45s, P3: 2s, P4: 15s, P5: 12s
Tumor	CosMx	0.8M	68	P1: 12s, P2: 33s, P3: 1s, P4: 12s, P5: 10s
Normal	Xenium	0.4M	24	P1: 5s, P2: 14s, P3: 1s
Normal	MERSCOPE	0.3M	18	P1: 4s, P2: 10s, P3: 1s

HDBSCAN clustering (Phase 2) dominates the processing time, scaling approximately as $O(n \log n)$ in the number of cells. The connectivity analysis (Phase 1) is the second-most expensive step due to the kd-tree pair query. Grid detection (Phase 4) and reassignment (Phase 5) are comparatively fast.

S4.2 Evaluation on TMA datasets

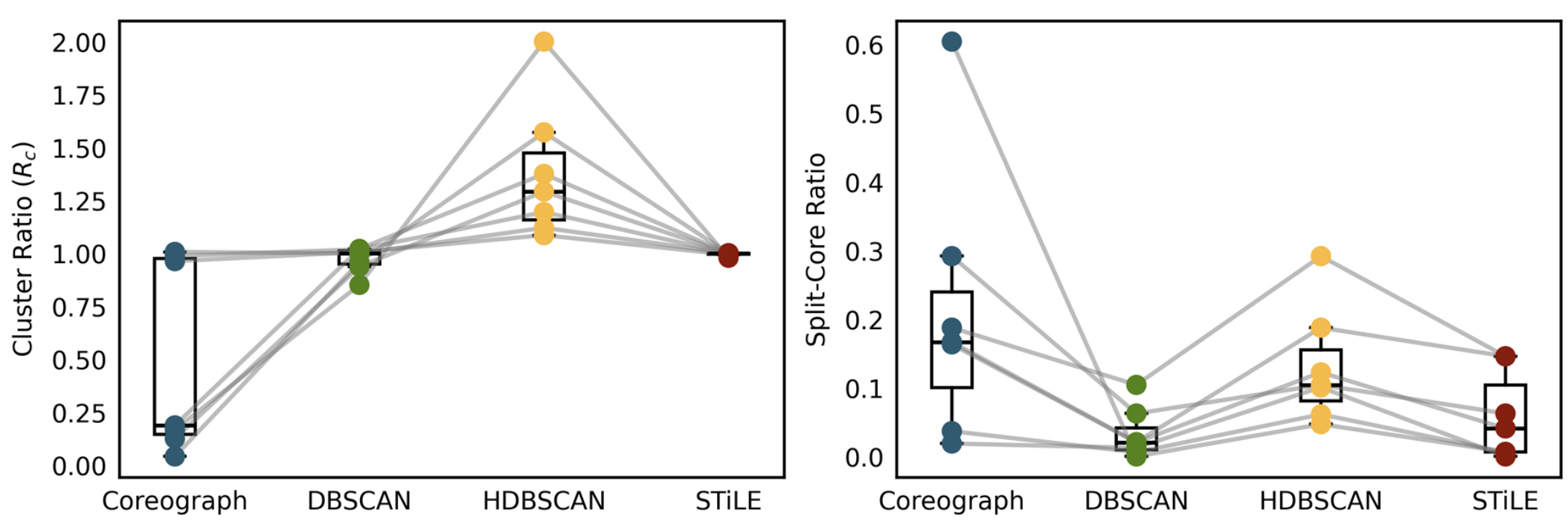

Figure S1: **STiLE recovers the correct number of cores, whereas baseline methods over- or under-segment.** Two complementary measures of over-segmentation for Coreograph, DBSCAN, HDBSCAN, and STiLE on the publicly available TMA datasets. Cluster Ratio (R_C, left) is the number of detected clusters divided by the number of expected cores, where R_C = 1 indicates exact one-to-one correspondence, $R_C > 1$ over-segmentation, and $R_C < 1$ incomplete core recovery. Split-Core Ratio (right) is the fraction of expected cores fragmented across two or more clusters (ideal = 0). Each point is one dataset; grey lines connect the same dataset across methods; boxplots show the distribution across datasets. Coreograph under-detects cores ($R_C << 1$), DBSCAN tends to merge multiple cores ($R_C < 1$), and HDBSCAN over-segments ($R_C > 1$) the cores. In contrast, STiLE achieves consistent scores with minimal over-segmentation $R_C = 1$, and low R_{SC} across all datasets.

S4.3 Simulation Benchmark

We systematically evaluated STiLE on 396 synthetic TMA datasets generated with the built-in simulation module. Datasets varied three difficulty axes-radius jitter fraction (0-100%, 11 levels), radial density bias (0-100%, 6 levels), and missing core fraction (0-50%, 6 levels) in a full factorial design. All datasets included elliptical cores, tissue slits (60% of cores), bubbles,

folds, affine warping (rotation + scale + shear), thin-plate-spline non-rigid deformation (amplitude 100), edge cropping, and block/random missingness.

- **Slits**: Linear cracks through cores, parameterized by probability, count, width, and angle.
- **Missing cores**: Random core dropout and block (row/column) dropout.
- **Bubbles**: Circular regions of cell removal, simulating air bubbles.
- **Folds**: Curved bands of cell removal, simulating tissue folds.
- **Affine deformations**: Global rotation, scaling, shear, and translation.
- **Thin-plate spline (TPS) warping**: Non-rigid deformation with configurable amplitude and control points.
- **Cell sampling**: Cells are sampled within each core from a Poisson distribution and center-biased density.
- **Grid configuration**: The number of rows and columns, inter-core pitch, core radius, and origin are pre-specified. Core positions are subject to center jitter, and core shapes can be elliptical with configurable eccentricity and orientation.

The simulation outputs cell coordinates with ground-truth core assignments, enabling automated evaluation via the metrics module.Tables S3-S5 report ARI stratified by each axis.

Table S3: ARI by radius jitter fraction. Each level contains 36 datasets.

**Jitter (%)**	n	**ARI mean**	**ARI min**
0	36	0.9946	0.9356
10	36	0.9987	0.9904
20	36	0.9988	0.9917
30	36	0.9925	0.9094
40	36	0.9928	0.9094
50	36	0.9880	0.9494
60	36	0.9833	0.8853
70	36	0.9919	0.9468
80	36	0.9901	0.9076
90	36	0.9921	0.9451
100	36	0.9905	0.9416

Table S4: ARI by radial density bias. Each level contains 66 datasets.

**Bias (%)**	n	**ARI mean**	**ARI min**
0	66	0.9925	0.9356
20	66	0.9919	0.9434
40	66	0.9937	0.9447
60	66	0.9941	0.9416
80	66	0.9936	0.9471
100	66	0.9869	0.8853

Table S5: ARI by missing core fraction. Each level contains 66 datasets.

**Missing (%)**	n	**ARI mean**	**ARI min**
0	66	0.9705	0.9369
10	66	0.9902	0.9055
20	66	1.0000	0.9995
30	66	1.0000	1.0000
40	66	0.9921	0.8853
50	66	1.0000	0.9992

Across all 396 datasets, STiLE achieved a mean ARI of 0.992 (median 1.000, minimum 0.885), with 395 of 396 datasets (99.7%) exceeding ARI 0.90. Performance remained consistently high across all jitter levels (mean ARI $\geq$ 0.983), density bias levels (mean ARI $\geq$ 0.987), and missingness levels (mean ARI $\geq$ 0.970).

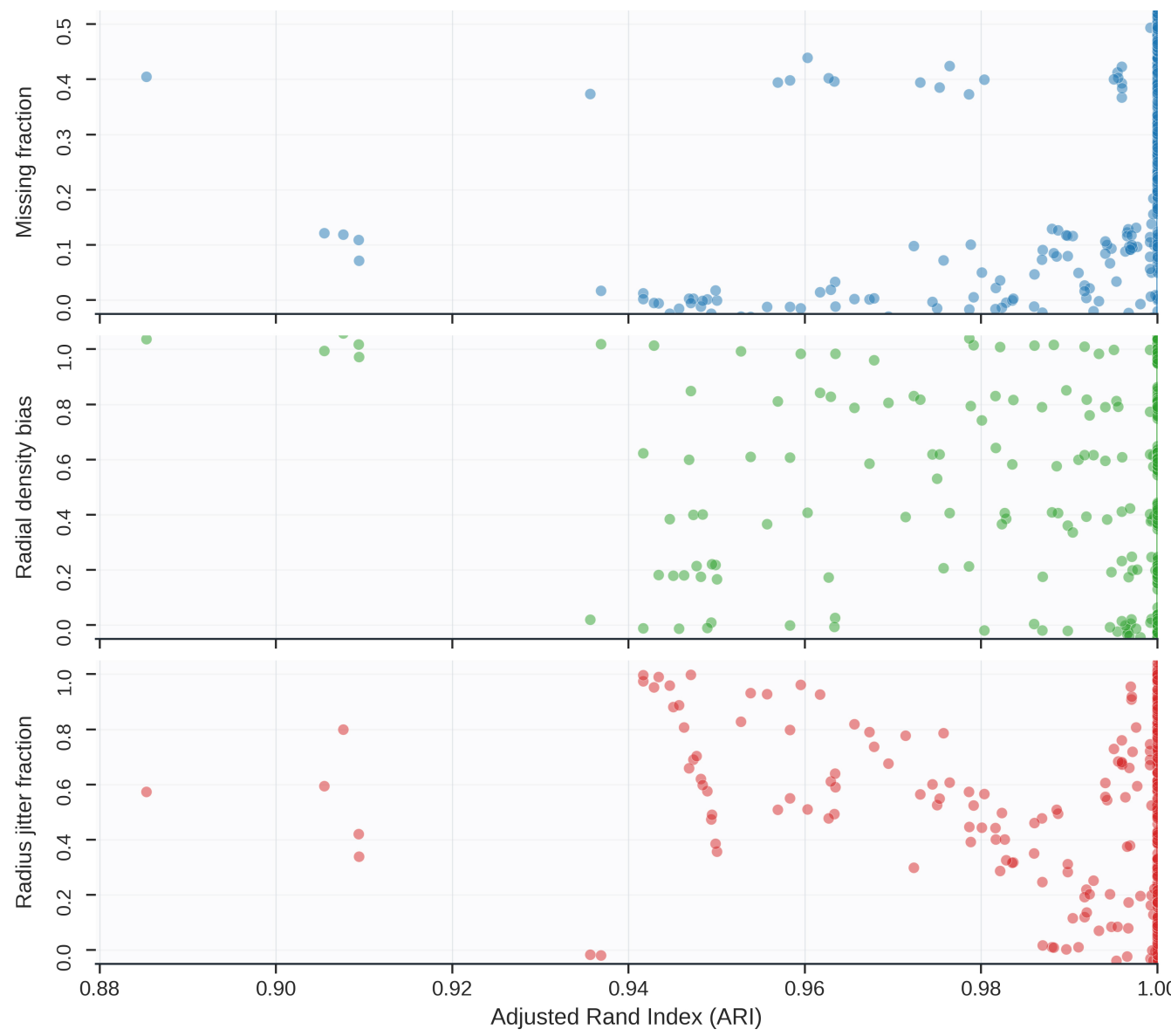

Figure S2: **Evaluation on simulated TMA datasets designed to mimic realistic TMA datasets**. Each simulated dataset contains elliptical cores with varying levels of controlled structural perturbations, including missing cores, radial density bias and radius jitter. Each point represents a single simulated dataset and the performance is measured using the Adjusted Rand Index (ARI)

S5 Data Format Conventions

S5.1 Input Requirements

STiLE accepts data in two formats:

- **AnnData** (`.h5ad`): Cell coordinates must be present as numeric columns in `adata.obs`. Default column names are `x_centroid` and `y_centroid`, configurable via `x_col` and `y_col` parameters.
- **CSV**: Must contain at least two numeric columns representing spatial coordinates. The user selects the appropriate columns in the Streamlit interface.

Platform-specific raw data can be converted to standardized format using `stile prepare_data`:

- **Vizgen MERSCOPE**: Reads `cell_by_gene.csv` and `cell_metadata.csv`.
- **10x Xenium**: Reads `cell_feature_matrix.h5` and `cells.csv`.
- **NanoString CosMx**: Reads `exprMat_file.csv` and `cell_metadata.csv`.

S6 Parameter Reference

Table S6 lists all user-adjustable parameters, their defaults in both the CLI and interactive Streamlit interface, and their effects on pipeline behavior.

Parameter guidance. For well-separated TMAs with 20-50 cores, the defaults generally suffice. For dense TMAs with >100 cores, we recommend: (i) reducing `peak_prominence` and `peak_distance` to detect closely spaced peaks, (ii) using the region-based workflow to limit peak detection to sub-regions with locally uniform spacing, and (iii) increasing `min_cluster_size` proportionally to the cell density to avoid over-fragmentation.

S6.1 Output Format

Core assignments are stored in `adata.obs` with the following columns:

- `hdbscan_cluster` (int): HDBSCAN cluster label (-1 = noise).
- `connected_component` (int): Connected component ID.
- `merged_cluster` (int): Component-merged cluster label (-1 = unassigned).
- `path_block_core` (str): Final core label, formatted as `core_{x}_{y}` where x and y are the integer coordinates of the assigned core center, or `unassigned`.

Table S6: Complete parameter reference.

**Parameter**	**Default**	
Phase 1: Connectivity		
`buffer_distance`	auto	Buffer radius (0 = median NN dist.)
Phase 2: HDBSCAN		
`min_cluster_size`	200	Min. points per cluster
`min_samples`	7	Neighborhood density threshold
Phase 4: Grid detection		
`peak_prominence`	25	Min. peak height vs. baseline
`peak_distance`	25	Min. inter-peak spacing (bins)
`density_bins_x`	200	Histogram resolution (x-axis)
`density_bins_y`	200	Histogram resolution (y-axis)
`max_x_peaks`	-	Max. detected x-peaks
`max_y_peaks`	-	Max. detected y-peaks
`min_cells_per_center`	10	Filter threshold for cores
General		
`rotation_deg`	0	Pre-processing rotation (degrees)
`x_col / y_col`	`x/y_centroid`	Coordinate column names

- `path_block_core_x`, `path_block_core_y` (float): Coordinates of the assigned core center.
- `core_name` (str, optional): Human-readable core name from layout mapping.

S7 Evaluation Metrics

- **Adjusted Rand Index (ARI)**: Measures agreement between predicted and true clusterings, corrected for chance. Range $[-1, 1]$; 1 indicates perfect agreement.
- **Homogeneity**: Each predicted cluster contains only members of a single true cluster.
- **Completeness**: All members of a true cluster are assigned to the same predicted cluster.
- **Weighted Majority Capture**: For each true cluster, the fraction of its cells assigned to the most common predicted cluster, weighted by cluster size. Values near 1 indicate low fragmentation.
- **The Cluster Ratio (R_C)**: the number of detected clusters divided by the number of expected cores, where a value of 1 denotes an exact one-to-one correspondence
- **The Split-Core Ratio (R_{SC})**: The fraction of expected cores fragmented across two or more clusters, where 0 denotes that no core is split.
